# Dichlorido{*N*-[2-(diphenylphosphanyl)benzylidene]-2,6-diisopropylaniline-κ^2^ *P*,*N*}platinum(II)

**DOI:** 10.1107/S1600536811040098

**Published:** 2011-10-05

**Authors:** Haleden Chiririwa, Reinout Meijboom

**Affiliations:** a Research Centre for Synthesis and Catalysis, Department of Chemistry, University of Johannesburg, PO Box 524 Auckland Park, Johannesburg 2006, South Africa

## Abstract

The title compound, [PtCl_2_(C_31_H_32_NP)], is a Pt^II^ complex with an NPCl_2_ coordination sphere in which the Pt^II^ atom is coordinated to the imino N and phosphane P atoms of the ligand and to two *cis* Cl ions, giving a slightly distorted square-planar geometry. The P—Pt—N angle is 89.80 (5)° and the corresponding angle between the Cl ions is 87.92 (2)°.

## Related literature

For related structures, see: Chiririwa *et al.* (2011 ▶); Ghilardi *et al.* (1992 ▶); Sanchez *et al.* (1998 ▶, 2001 ▶). 
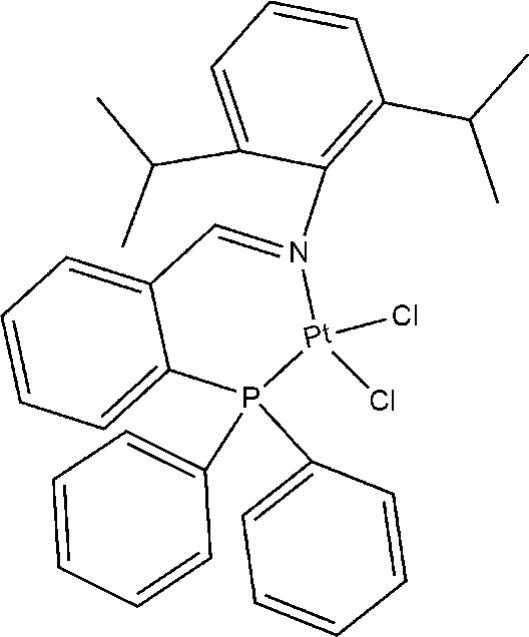


## Experimental

### 

#### Crystal data

[PtCl_2_(C_31_H_32_NP)]
*M* *_r_* = 715.54
Monoclinic, 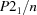

*a* = 12.0686 (7) Å
*b* = 13.4007 (7) Å
*c* = 17.8182 (10) Å
β = 105.819 (1)°
*V* = 2772.6 (3) Å^3^
*Z* = 4
Mo *K*α radiation
μ = 5.33 mm^−1^
*T* = 173 K
0.22 × 0.11 × 0.09 mm

#### Data collection

Bruker Kappa DUO APEXII diffractometer
Absorption correction: multi-scan (*SADABS*; Bruker, 2007 ▶) *T*_min_ = 0.587, *T*_max_ = 0.746
40326 measured reflections
7788 independent reflections
7035 reflections with *I* > 2σ(*I*)
*R*_int_ = 0.051

#### Refinement

*R*[*F* ^2^ > 2σ(*F* ^2^)] = 0.021
*wR*(*F* ^2^) = 0.051
*S* = 1.06
7788 reflections
326 parameters
H-atom parameters constrained
Δρ_max_ = 0.96 e Å^−3^
Δρ_min_ = −0.80 e Å^−3^

### 

Data collection: *APEX2* (Bruker, 2007 ▶); cell refinement: *SAINT* (Bruker, 2007 ▶); data reduction: *SAINT*; program(s) used to solve structure: *SHELXS97* (Sheldrick, 2008 ▶); program(s) used to refine structure: *SHELXL97* (Sheldrick, 2008 ▶); molecular graphics: *DIAMOND* (Brandenburg & Putz, 2005 ▶) and *ORTEP-3* (Farrugia, 1997 ▶); software used to prepare material for publication: *WinGX* (Farrugia, 1999 ▶).

## Supplementary Material

Crystal structure: contains datablock(s) global, I. DOI: 10.1107/S1600536811040098/go2029sup1.cif

Structure factors: contains datablock(s) I. DOI: 10.1107/S1600536811040098/go2029Isup2.hkl

Additional supplementary materials:  crystallographic information; 3D view; checkCIF report

## Figures and Tables

**Table 1:** Selected bond lengths (Å)

Pt1—N1	2.0421 (18)
Pt1—P1	2.2128 (6)
Pt1—Cl2	2.2901 (6)
Pt1—Cl1	2.3512 (6)

**Fig. 1.**
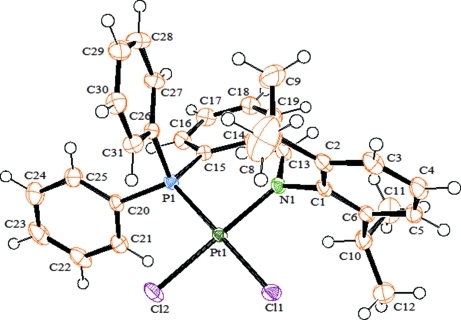
View of (I) (35% probability displacement ellipsoids).

## supplementary crystallographic information

### Comment

In recent years, platinum complexes with iminophosphane ligands of the *N*-[(2-diphenylphosphanyl)benzylidene]amine type have been used as catalysts (or catalyst precursors) in a variety organic reactions. To the best of our knowledge, no structures have been determined so far, concerning the free ligand -(2-(diphenylphosphanyl)benzylidene) -2,6-diisopropylbenzenamine, where the potentially bidentate ligand is chelated to the metal through the phosphorus and imino nitrogen atoms (Fig. 1). The platinum complex has a distorted square planar geometry with the P–Pt–N angle of 89.80 (5)° and the corresponding angle between the chloride ligands of 87.92 (2)°. Selected bond lengths are given in Table 1.

### Experimental

To a dry CH_2_Cl_2_ (10 ml) solution of the precursor [Pt(COD)Cl_2_] was added an equimolar amount of (2-(diphenylphosphanyl)benzylidene) -2,6-diisopropylbenzenamine in CH_2_Cl_2_ (10 ml) solution and the reaction was stirred at room temperature for 1 hr. The yellow solution was concentrated under reduced pressure to half volume and the addition of *ca* 10 ml hexane caused precipitation of the complex, which was filtered off, washed with Et_2_O and dried under vacuum for 4 hours. Yellow crystals used in the X-ray diffraction studies were grown by slow evaporation of a solution of the compound in a CH_2_Cl_2_-hexane solution at room temperature.

### Refinement

The methyl, methine and aromatic H atoms were placed in geometrically idealized positions and constrained to ride on their parent atoms, with C—H = 0.95 Å for aromatic, C—H = 0.99 Å for ^i^Pr CH, C—H = 0.95 Å for CH and C—H = 0.98 for Me groups.

### Crystal data

**Table:**

[PtCl_2_(C_31_H_32_NP)]	*F*(000) = 1408
*M**_r_* = 715.54	*D*_x_ = 1.714 Mg m^−^^3^
Monoclinic, *P*2_1_/*n*	Mo *K*α radiation, λ = 0.71073 Å
Hall symbol: -P 2yn	Cell parameters from 40326 reflections
*a* = 12.0686 (7) Å	θ = 1.8–29.6°
*b* = 13.4007 (7) Å	µ = 5.33 mm^−^^1^
*c* = 17.8182 (10) Å	*T* = 173 K
β = 105.819 (1)°	Diamond, yellow
*V* = 2772.6 (3) Å^3^	0.22 × 0.11 × 0.09 mm
*Z* = 4	

### Data collection

**Table:**

Bruker Kappa DUO APEXII diffractometer	7788 independent reflections
Radiation source: fine-focus sealed tube	7035 reflections with *I* > 2σ(*I*)
graphite	*R*_int_ = 0.051
0.5° φ scans and ω scans	θ_max_ = 29.6°, θ_min_ = 1.8°
Absorption correction: multi-scan (*SADABS*; Bruker, 2007)	*h* = −16→16
*T*_min_ = 0.587, *T*_max_ = 0.746	*k* = −18→18
40326 measured reflections	*l* = −24→24

### Refinement

**Table:**

Refinement on *F*^2^	Secondary atom site location: difference Fourier map
Least-squares matrix: full	Hydrogen site location: inferred from neighbouring sites
*R*[*F*^2^ > 2σ(*F*^2^)] = 0.021	H-atom parameters constrained
*wR*(*F*^2^) = 0.051	*w* = 1/[σ^2^(*F*_o_^2^) + (0.0135*P*)^2^ + 1.4942*P*] where *P* = (*F*_o_^2^ + 2*F*_c_^2^)/3
*S* = 1.06	(Δ/σ)_max_ = 0.004
7788 reflections	Δρ_max_ = 0.96 e Å^−^^3^
326 parameters	Δρ_min_ = −0.80 e Å^−^^3^
0 restraints	Extinction correction: *SHELXL97* (Sheldrick, 2008), Fc^*^=kFc[1+0.001xFc^2^λ^3^/sin(2θ)]^-1/4^
Primary atom site location: structure-invariant direct methods	Extinction coefficient: 0.00096 (7)

### Special details

**Table:**

Experimental. Half sphere of data collected using SAINT strategy (Bruker, 2007). Crystal to detector distance = 50mm; combination of φ and ω scans of 0.5°, 70s per °, 2 iterations.
Geometry. All esds (except the esd in the dihedral angle between two l.s. planes) are estimated using the full covariance matrix. The cell esds are taken into account individually in the estimation of esds in distances, angles and torsion angles; correlations between esds in cell parameters are only used when they are defined by crystal symmetry. An approximate (isotropic) treatment of cell esds is used for estimating esds involving l.s. planes.
Refinement. Refinement of F^2^ against ALL reflections. The weighted R-factor wR and goodness of fit S are based on F^2^, conventional R-factors R are based on F, with F set to zero for negative F^2^. The threshold expression of F^2^ > 2sigma(F^2^) is used only for calculating R-factors(gt) etc. and is not relevant to the choice of reflections for refinement. R-factors based on F^2^ are statistically about twice as large as those based on F, and R- factors based on ALL data will be even larger.

### Fractional atomic coordinates and isotropic or equivalent isotropic displacement parameters (Å^2^)

**Table:**

	*x*	*y*	*z*	*U*_iso_*/*U*_eq_	
Pt1	0.471492 (7)	0.192010 (6)	0.184668 (4)	0.01548 (3)	
Cl1	0.62398 (5)	0.26159 (4)	0.28210 (3)	0.02483 (12)	
Cl2	0.42312 (6)	0.10259 (4)	0.28063 (3)	0.02814 (13)	
P1	0.31657 (5)	0.13788 (4)	0.09630 (3)	0.01714 (11)	
N1	0.51653 (16)	0.27364 (14)	0.10076 (11)	0.0182 (4)	
C1	0.5937 (2)	0.35990 (16)	0.12390 (12)	0.0191 (4)	
C2	0.5466 (2)	0.45114 (16)	0.13757 (13)	0.0216 (5)	
C3	0.6216 (2)	0.53261 (17)	0.15398 (14)	0.0267 (5)	
H3	0.5930	0.5961	0.1634	0.032*	
C4	0.7369 (2)	0.52253 (18)	0.15679 (14)	0.0285 (5)	
H4	0.7861	0.5791	0.1671	0.034*	
C5	0.7805 (2)	0.43054 (19)	0.14464 (13)	0.0261 (5)	
H5	0.8598	0.4245	0.1471	0.031*	
C6	0.7099 (2)	0.34605 (17)	0.12885 (13)	0.0210 (4)	
C7	0.4206 (2)	0.46235 (18)	0.13395 (14)	0.0253 (5)	
H7	0.3927	0.3953	0.1456	0.030*	
C8	0.3995 (3)	0.5340 (4)	0.1941 (3)	0.0731 (13)	
H8A	0.3167	0.5377	0.1892	0.110*	
H8B	0.4281	0.6004	0.1857	0.110*	
H8C	0.4400	0.5104	0.2465	0.110*	
C9	0.3502 (3)	0.4911 (4)	0.0535 (2)	0.0679 (13)	
H9A	0.2692	0.4977	0.0531	0.102*	
H9B	0.3576	0.4394	0.0163	0.102*	
H9C	0.3780	0.5548	0.0388	0.102*	
C10	0.7599 (2)	0.24529 (18)	0.11751 (16)	0.0278 (5)	
H10	0.7002	0.1937	0.1182	0.033*	
C11	0.7861 (3)	0.2384 (2)	0.03825 (18)	0.0405 (7)	
H11A	0.8181	0.1724	0.0327	0.061*	
H11B	0.8420	0.2900	0.0348	0.061*	
H11C	0.7149	0.2482	−0.0035	0.061*	
C12	0.8663 (3)	0.2210 (2)	0.18381 (19)	0.0381 (7)	
H12A	0.8964	0.1556	0.1746	0.057*	
H12B	0.8457	0.2198	0.2333	0.057*	
H12C	0.9253	0.2721	0.1862	0.057*	
C13	0.4897 (2)	0.25884 (16)	0.02661 (13)	0.0208 (4)	
H13	0.5177	0.3078	−0.0022	0.025*	
C14	0.4229 (2)	0.17853 (16)	−0.02034 (13)	0.0197 (4)	
C15	0.3459 (2)	0.11545 (16)	0.00345 (13)	0.0186 (4)	
C16	0.2884 (2)	0.04182 (18)	−0.04764 (14)	0.0256 (5)	
H16	0.2366	−0.0017	−0.0321	0.031*	
C17	0.3059 (2)	0.03122 (18)	−0.12132 (14)	0.0278 (5)	
H17	0.2662	−0.0195	−0.1555	0.033*	
C18	0.3803 (2)	0.09354 (19)	−0.14480 (14)	0.0279 (5)	
H18	0.3924	0.0860	−0.1950	0.033*	
C19	0.4373 (2)	0.16743 (19)	−0.09493 (14)	0.0251 (5)	
H19	0.4873	0.2115	−0.1118	0.030*	
C20	0.2426 (2)	0.02665 (16)	0.11437 (13)	0.0206 (4)	
C21	0.3064 (2)	−0.05910 (17)	0.14187 (14)	0.0257 (5)	
H21	0.3880	−0.0578	0.1532	0.031*	
C22	0.2507 (3)	−0.14614 (19)	0.15259 (15)	0.0334 (6)	
H22	0.2944	−0.2046	0.1709	0.040*	
C23	0.1333 (3)	−0.1486 (2)	0.13708 (17)	0.0422 (8)	
H23	0.0956	−0.2081	0.1455	0.051*	
C24	0.0704 (3)	−0.0652 (2)	0.10942 (19)	0.0446 (8)	
H24	−0.0112	−0.0676	0.0980	0.053*	
C25	0.1236 (2)	0.0233 (2)	0.09763 (16)	0.0333 (6)	
H25	0.0789	0.0808	0.0783	0.040*	
C26	0.2095 (2)	0.23674 (16)	0.07734 (13)	0.0196 (4)	
C27	0.1482 (2)	0.26547 (18)	0.00241 (14)	0.0251 (5)	
H27	0.1604	0.2324	−0.0419	0.030*	
C28	0.0687 (2)	0.3433 (2)	−0.00702 (16)	0.0327 (6)	
H28	0.0259	0.3632	−0.0578	0.039*	
C29	0.0523 (2)	0.39161 (18)	0.05787 (16)	0.0304 (6)	
H29	−0.0014	0.4449	0.0512	0.036*	
C30	0.1133 (2)	0.36307 (19)	0.13237 (15)	0.0278 (5)	
H30	0.1011	0.3963	0.1765	0.033*	
C31	0.1923 (2)	0.28549 (19)	0.14221 (15)	0.0253 (5)	
H31	0.2346	0.2657	0.1932	0.030*	

### Atomic displacement parameters (Å^2^)

**Table:**

	*U*^11^	*U*^22^	*U*^33^	*U*^12^	*U*^13^	*U*^23^
Pt1	0.01852 (5)	0.01268 (5)	0.01450 (5)	−0.00153 (3)	0.00323 (3)	0.00084 (3)
Cl1	0.0269 (3)	0.0241 (3)	0.0190 (2)	−0.0066 (2)	−0.0012 (2)	0.0021 (2)
Cl2	0.0395 (4)	0.0264 (3)	0.0181 (2)	−0.0118 (2)	0.0071 (2)	0.0031 (2)
P1	0.0194 (3)	0.0156 (2)	0.0164 (2)	−0.0027 (2)	0.0048 (2)	−0.00019 (19)
N1	0.0191 (10)	0.0161 (8)	0.0192 (9)	−0.0034 (7)	0.0049 (7)	0.0000 (7)
C1	0.0258 (12)	0.0157 (10)	0.0152 (9)	−0.0066 (8)	0.0045 (9)	0.0002 (7)
C2	0.0286 (13)	0.0181 (10)	0.0179 (10)	−0.0049 (9)	0.0060 (9)	0.0012 (8)
C3	0.0395 (15)	0.0166 (10)	0.0241 (11)	−0.0061 (10)	0.0090 (11)	−0.0033 (9)
C4	0.0350 (15)	0.0222 (12)	0.0256 (12)	−0.0141 (10)	0.0036 (11)	−0.0019 (9)
C5	0.0258 (13)	0.0296 (12)	0.0216 (11)	−0.0089 (10)	0.0039 (9)	0.0016 (9)
C6	0.0239 (12)	0.0203 (11)	0.0176 (10)	−0.0046 (9)	0.0035 (9)	0.0019 (8)
C7	0.0319 (14)	0.0189 (11)	0.0281 (12)	−0.0026 (9)	0.0131 (10)	0.0020 (9)
C8	0.050 (2)	0.097 (3)	0.080 (3)	−0.003 (2)	0.031 (2)	−0.046 (3)
C9	0.0313 (19)	0.123 (4)	0.046 (2)	0.002 (2)	0.0044 (15)	0.037 (2)
C10	0.0260 (13)	0.0217 (11)	0.0360 (13)	−0.0026 (9)	0.0090 (11)	0.0016 (10)
C11	0.0454 (18)	0.0368 (16)	0.0427 (16)	−0.0022 (13)	0.0176 (14)	−0.0119 (13)
C12	0.0305 (15)	0.0351 (14)	0.0480 (17)	0.0061 (12)	0.0092 (13)	0.0092 (13)
C13	0.0257 (12)	0.0183 (10)	0.0187 (10)	−0.0046 (9)	0.0064 (9)	0.0009 (8)
C14	0.0230 (12)	0.0166 (10)	0.0191 (10)	−0.0004 (8)	0.0050 (9)	−0.0014 (8)
C15	0.0200 (11)	0.0171 (10)	0.0181 (10)	0.0008 (8)	0.0041 (8)	−0.0014 (8)
C16	0.0298 (13)	0.0209 (11)	0.0249 (11)	−0.0057 (9)	0.0057 (10)	−0.0039 (9)
C17	0.0357 (15)	0.0221 (12)	0.0233 (11)	−0.0017 (10)	0.0044 (10)	−0.0070 (9)
C18	0.0346 (14)	0.0301 (13)	0.0188 (11)	0.0010 (10)	0.0072 (10)	−0.0049 (9)
C19	0.0293 (14)	0.0269 (11)	0.0199 (11)	−0.0042 (10)	0.0082 (10)	−0.0012 (9)
C20	0.0245 (12)	0.0192 (10)	0.0188 (10)	−0.0076 (9)	0.0072 (9)	−0.0006 (8)
C21	0.0313 (14)	0.0210 (11)	0.0261 (12)	−0.0031 (9)	0.0099 (10)	−0.0012 (9)
C22	0.0567 (19)	0.0184 (12)	0.0261 (12)	−0.0084 (11)	0.0128 (12)	0.0002 (9)
C23	0.059 (2)	0.0322 (15)	0.0345 (15)	−0.0241 (14)	0.0123 (14)	0.0024 (12)
C24	0.0329 (16)	0.0485 (18)	0.0491 (18)	−0.0213 (14)	0.0059 (14)	0.0085 (14)
C25	0.0273 (14)	0.0326 (14)	0.0374 (14)	−0.0091 (11)	0.0043 (11)	0.0067 (11)
C26	0.0181 (11)	0.0195 (10)	0.0207 (10)	−0.0029 (8)	0.0043 (9)	0.0007 (8)
C27	0.0307 (14)	0.0231 (12)	0.0194 (11)	−0.0001 (10)	0.0036 (10)	0.0004 (9)
C28	0.0356 (16)	0.0285 (13)	0.0296 (13)	0.0064 (11)	0.0015 (11)	0.0069 (10)
C29	0.0320 (14)	0.0214 (11)	0.0389 (14)	0.0068 (10)	0.0115 (12)	0.0057 (10)
C30	0.0291 (14)	0.0256 (12)	0.0310 (13)	0.0026 (10)	0.0120 (11)	−0.0023 (10)
C31	0.0256 (13)	0.0262 (11)	0.0234 (11)	0.0014 (10)	0.0055 (10)	−0.0017 (9)

### Geometric parameters (Å, °)

**Table:**

Pt1—N1	2.0421 (18)	C12—H12B	0.9800
Pt1—P1	2.2128 (6)	C12—H12C	0.9800
Pt1—Cl2	2.2901 (6)	C13—C14	1.463 (3)
Pt1—Cl1	2.3512 (6)	C13—H13	0.9500
P1—C15	1.809 (2)	C14—C19	1.394 (3)
P1—C20	1.811 (2)	C14—C15	1.405 (3)
P1—C26	1.816 (2)	C15—C16	1.392 (3)
N1—C13	1.287 (3)	C16—C17	1.393 (3)
N1—C1	1.471 (3)	C16—H16	0.9500
C1—C6	1.393 (3)	C17—C18	1.372 (4)
C1—C2	1.398 (3)	C17—H17	0.9500
C2—C3	1.398 (3)	C18—C19	1.382 (3)
C2—C7	1.511 (4)	C18—H18	0.9500
C3—C4	1.385 (4)	C19—H19	0.9500
C3—H3	0.9500	C20—C25	1.385 (4)
C4—C5	1.381 (4)	C20—C21	1.396 (3)
C4—H4	0.9500	C21—C22	1.385 (3)
C5—C6	1.399 (3)	C21—H21	0.9500
C5—H5	0.9500	C22—C23	1.369 (4)
C6—C10	1.515 (3)	C22—H22	0.9500
C7—C9	1.503 (4)	C23—C24	1.366 (5)
C7—C8	1.512 (4)	C23—H23	0.9500
C7—H7	1.0000	C24—C25	1.392 (4)
C8—H8A	0.9800	C24—H24	0.9500
C8—H8B	0.9800	C25—H25	0.9500
C8—H8C	0.9800	C26—C31	1.392 (3)
C9—H9A	0.9800	C26—C27	1.393 (3)
C9—H9B	0.9800	C27—C28	1.396 (4)
C9—H9C	0.9800	C27—H27	0.9500
C10—C12	1.525 (4)	C28—C29	1.385 (4)
C10—C11	1.532 (4)	C28—H28	0.9500
C10—H10	1.0000	C29—C30	1.385 (4)
C11—H11A	0.9800	C29—H29	0.9500
C11—H11B	0.9800	C30—C31	1.390 (3)
C11—H11C	0.9800	C30—H30	0.9500
C12—H12A	0.9800	C31—H31	0.9500
			
N1—Pt1—P1	89.80 (5)	C10—C12—H12A	109.5
N1—Pt1—Cl2	178.85 (5)	C10—C12—H12B	109.5
P1—Pt1—Cl2	91.25 (2)	H12A—C12—H12B	109.5
N1—Pt1—Cl1	90.98 (5)	C10—C12—H12C	109.5
P1—Pt1—Cl1	174.20 (2)	H12A—C12—H12C	109.5
Cl2—Pt1—Cl1	87.92 (2)	H12B—C12—H12C	109.5
C15—P1—C20	104.75 (10)	N1—C13—C14	130.2 (2)
C15—P1—C26	104.92 (10)	N1—C13—H13	114.9
C20—P1—C26	105.85 (11)	C14—C13—H13	114.9
C15—P1—Pt1	111.56 (8)	C19—C14—C15	119.2 (2)
C20—P1—Pt1	120.35 (8)	C19—C14—C13	115.6 (2)
C26—P1—Pt1	108.26 (7)	C15—C14—C13	125.2 (2)
C13—N1—C1	111.82 (18)	C16—C15—C14	118.8 (2)
C13—N1—Pt1	128.78 (16)	C16—C15—P1	121.94 (18)
C1—N1—Pt1	119.35 (13)	C14—C15—P1	119.10 (16)
C6—C1—C2	123.8 (2)	C15—C16—C17	120.8 (2)
C6—C1—N1	117.5 (2)	C15—C16—H16	119.6
C2—C1—N1	118.7 (2)	C17—C16—H16	119.6
C3—C2—C1	116.6 (2)	C18—C17—C16	120.4 (2)
C3—C2—C7	121.2 (2)	C18—C17—H17	119.8
C1—C2—C7	122.1 (2)	C16—C17—H17	119.8
C4—C3—C2	121.2 (2)	C17—C18—C19	119.5 (2)
C4—C3—H3	119.4	C17—C18—H18	120.2
C2—C3—H3	119.4	C19—C18—H18	120.2
C5—C4—C3	120.2 (2)	C18—C19—C14	121.3 (2)
C5—C4—H4	119.9	C18—C19—H19	119.3
C3—C4—H4	119.9	C14—C19—H19	119.3
C4—C5—C6	121.1 (2)	C25—C20—C21	119.3 (2)
C4—C5—H5	119.4	C25—C20—P1	121.37 (19)
C6—C5—H5	119.4	C21—C20—P1	119.20 (19)
C1—C6—C5	116.9 (2)	C22—C21—C20	119.9 (3)
C1—C6—C10	122.7 (2)	C22—C21—H21	120.0
C5—C6—C10	120.3 (2)	C20—C21—H21	120.0
C9—C7—C2	111.5 (2)	C23—C22—C21	120.5 (3)
C9—C7—C8	110.7 (3)	C23—C22—H22	119.8
C2—C7—C8	113.2 (2)	C21—C22—H22	119.8
C9—C7—H7	107.0	C24—C23—C22	119.8 (3)
C2—C7—H7	107.0	C24—C23—H23	120.1
C8—C7—H7	107.0	C22—C23—H23	120.1
C7—C8—H8A	109.5	C23—C24—C25	121.2 (3)
C7—C8—H8B	109.5	C23—C24—H24	119.4
H8A—C8—H8B	109.5	C25—C24—H24	119.4
C7—C8—H8C	109.5	C20—C25—C24	119.3 (3)
H8A—C8—H8C	109.5	C20—C25—H25	120.4
H8B—C8—H8C	109.5	C24—C25—H25	120.4
C7—C9—H9A	109.5	C31—C26—C27	120.4 (2)
C7—C9—H9B	109.5	C31—C26—P1	116.58 (18)
H9A—C9—H9B	109.5	C27—C26—P1	123.03 (18)
C7—C9—H9C	109.5	C26—C27—C28	119.3 (2)
H9A—C9—H9C	109.5	C26—C27—H27	120.3
H9B—C9—H9C	109.5	C28—C27—H27	120.3
C6—C10—C12	111.6 (2)	C29—C28—C27	119.9 (2)
C6—C10—C11	111.5 (2)	C29—C28—H28	120.1
C12—C10—C11	111.0 (2)	C27—C28—H28	120.1
C6—C10—H10	107.5	C30—C29—C28	120.8 (2)
C12—C10—H10	107.5	C30—C29—H29	119.6
C11—C10—H10	107.5	C28—C29—H29	119.6
C10—C11—H11A	109.5	C29—C30—C31	119.6 (2)
C10—C11—H11B	109.5	C29—C30—H30	120.2
H11A—C11—H11B	109.5	C31—C30—H30	120.2
C10—C11—H11C	109.5	C30—C31—C26	120.0 (2)
H11A—C11—H11C	109.5	C30—C31—H31	120.0
H11B—C11—H11C	109.5	C26—C31—H31	120.0
			
N1—Pt1—P1—C15	37.61 (9)	C13—C14—C15—P1	4.6 (3)
Cl2—Pt1—P1—C15	−142.85 (8)	C20—P1—C15—C16	17.3 (2)
N1—Pt1—P1—C20	160.88 (11)	C26—P1—C15—C16	−94.0 (2)
Cl2—Pt1—P1—C20	−19.57 (9)	Pt1—P1—C15—C16	149.04 (18)
N1—Pt1—P1—C26	−77.35 (10)	C20—P1—C15—C14	−167.23 (18)
Cl2—Pt1—P1—C26	102.20 (8)	C26—P1—C15—C14	81.5 (2)
P1—Pt1—N1—C13	−25.1 (2)	Pt1—P1—C15—C14	−35.5 (2)
P1—Pt1—N1—C1	157.67 (16)	C14—C15—C16—C17	−0.6 (4)
Cl1—Pt1—N1—C1	−16.58 (16)	P1—C15—C16—C17	174.92 (19)
C13—N1—C1—C6	−78.8 (3)	C15—C16—C17—C18	−0.1 (4)
Pt1—N1—C1—C6	98.9 (2)	C16—C17—C18—C19	−0.3 (4)
C13—N1—C1—C2	99.5 (2)	C17—C18—C19—C14	1.5 (4)
Pt1—N1—C1—C2	−82.8 (2)	C15—C14—C19—C18	−2.2 (4)
C6—C1—C2—C3	2.6 (3)	C13—C14—C19—C18	179.2 (2)
N1—C1—C2—C3	−175.59 (19)	C15—P1—C20—C25	−98.0 (2)
C6—C1—C2—C7	−178.1 (2)	C26—P1—C20—C25	12.6 (2)
N1—C1—C2—C7	3.7 (3)	Pt1—P1—C20—C25	135.53 (19)
C1—C2—C3—C4	−0.3 (3)	C15—P1—C20—C21	78.6 (2)
C7—C2—C3—C4	−179.6 (2)	C26—P1—C20—C21	−170.82 (18)
C2—C3—C4—C5	−1.1 (4)	Pt1—P1—C20—C21	−47.9 (2)
C3—C4—C5—C6	0.4 (4)	C25—C20—C21—C22	−0.4 (4)
C2—C1—C6—C5	−3.3 (3)	P1—C20—C21—C22	−177.04 (19)
N1—C1—C6—C5	174.91 (19)	C20—C21—C22—C23	−0.6 (4)
C2—C1—C6—C10	177.0 (2)	C21—C22—C23—C24	1.2 (4)
N1—C1—C6—C10	−4.8 (3)	C22—C23—C24—C25	−0.9 (5)
C4—C5—C6—C1	1.7 (3)	C21—C20—C25—C24	0.7 (4)
C4—C5—C6—C10	−178.6 (2)	P1—C20—C25—C24	177.3 (2)
C3—C2—C7—C9	88.2 (3)	C23—C24—C25—C20	−0.1 (5)
C1—C2—C7—C9	−91.0 (3)	C15—P1—C26—C31	−164.60 (19)
C3—C2—C7—C8	−37.4 (4)	C20—P1—C26—C31	84.9 (2)
C1—C2—C7—C8	143.4 (3)	Pt1—P1—C26—C31	−45.4 (2)
C1—C6—C10—C12	−129.7 (2)	C15—P1—C26—C27	14.0 (2)
C5—C6—C10—C12	50.7 (3)	C20—P1—C26—C27	−96.4 (2)
C1—C6—C10—C11	105.5 (3)	Pt1—P1—C26—C27	133.28 (19)
C5—C6—C10—C11	−74.2 (3)	C31—C26—C27—C28	−0.4 (4)
C1—N1—C13—C14	175.1 (2)	P1—C26—C27—C28	−179.0 (2)
Pt1—N1—C13—C14	−2.3 (4)	C26—C27—C28—C29	0.6 (4)
N1—C13—C14—C19	−161.8 (3)	C27—C28—C29—C30	−0.6 (4)
N1—C13—C14—C15	19.7 (4)	C28—C29—C30—C31	0.4 (4)
C19—C14—C15—C16	1.7 (3)	C29—C30—C31—C26	−0.2 (4)
C13—C14—C15—C16	−179.8 (2)	C27—C26—C31—C30	0.2 (4)
C19—C14—C15—P1	−173.92 (18)	P1—C26—C31—C30	178.9 (2)
